# Draft Genome Sequence of an Algicidal Bacterium, *Arenibacter* sp. Strain 6A1, Isolated from Seawater during an Akashiwo sanguinea Bloom in Shenzhen, China

**DOI:** 10.1128/MRA.00964-20

**Published:** 2020-12-10

**Authors:** Jiahuan Chen, Jing Tong, Shuangfei Li, Zhangli Hu, Liyan Wang, Huirong Chen

**Affiliations:** a Guangdong Technology Research Center for Marine Algal Bioengineering, Guangdong Key Laboratory of Plant Epigenetics, College of Life Sciences and Oceanography, Shenzhen University, Shenzhen, People’s Republic of China; b Shenzhen Engineering Laboratory for Marine Algal Biotechnology, Longhua Innovation Institute for Biotechnology, Shenzhen University, Shenzhen, People’s Republic of China; c Southern Marine Science and Engineering Guangdong Laboratory, Guangzhou, People’s Republic of China; University of Southern California

## Abstract

*Arenibacter* sp. strain 6A1 was isolated from seawater during an Akashiwo sanguinea bloom in Shenzhen, China. Here, we present its 4,666,208-bp genome sequence, consisting of 3,623 coding sequences with a G+C content of 38.95%.

## ANNOUNCEMENT

Harmful algal blooms (HABs), as the consequence of eutrophication, are causing great damage to fisheries, tourism, and public health and disrupting the ecological balance (1). It has been discovered that some marine bacteria play a significant role in the demise of HABs (1). Some marine bacteria can not only inhibit algal growth but also lyse algal cells directly or indirectly (2, 3). *Arenibacter* sp. strain 6A1 was isolated and identified from surface water during a dinoflagellate bloom caused by Akashiwo sanguinea in the eastern coastal area of Shenzhen, China (22°34.5′N, 114°31.27′E; 0.5 m depth). Seawater recovered during an *A. sanguinea* bloom was filtered through a filtration membrane with 0.22-μm pore size, and then the membrane was put into 2216E medium (per liter of seawater, 0.1 g of ferric phosphate, 1 g of yeast extract, 5 g of peptone) for cultivation. *Arenibacter* sp. strain 6A1 was sampled from a colony formed after multiple-dilution streaking onto marine agar 2216E.

A single colony of strain 6A1 was initially identified by phylogenetic analysis of its 16S rRNA gene, which was amplified from lysed colony material by PCR with the universal primer pair 27F (5′-AGAGTTTGATCCTGGCTCAG-3′) and 1492R (5′-GGTTACCTTGTTACGACTT-3′) (4). Sanger sequencing of the amplicon and comparison of its sequence with the sequences of type strains by means of BLASTn (5) revealed its genus as *Arenibacter* (GenBank accession number MG396982). The colony was reinoculated into 100 ml 2216E medium and incubated at 30°C (6) before DNA extraction.

Genomic DNA was extracted from strain 6A1 using a bacterial DNA kit (OMEGA, USA) following the manufacturer’s instructions. A paired-end sequencing library was prepared using the TruSeq Nano DNA library prep kit. The Illumina HiSeq platform was used for sequencing (7) the paired-end library, with a read length of 2 × 150 bp. The constructed library was quantified by Qubit 3.0, and the insert size was detected using an Agilent 2100 instrument (8). Illumina sequencing was performed at Shenzhen Realomics Biotech Co., Ltd. The raw data were filtered using fastp v.0.13.1 (9) to obtain clean data and assembled using SOAPdenovo v.2.04) with default parameters. GapCloser v.1.12 was used to close the gaps. Default parameters were used for all software unless otherwise specified. The draft genome sequence comprises 60 contigs from 1,670,000 reads with a mean coverage of 100× and an *N*_50_ value of 261,079 bp.

The assembly was annotated with NCBI Prokaryotic Genome Annotation Pipeline (PGAP) v.4.11 (10), which produced a 4,666,208-bp genome sequence with an average G+C content of 38.95%. This inferred 3,623 coding sequences, 32 pseudogenes, 57 tRNAs, 4 rRNAs, and 4 noncoding RNAs.

### Data availability.

This whole-genome sequencing project has been deposited in GenBank under accession number JAAXCQ000000000.1, BioProject accession number PRJNA614631, and BioSample accession number SAMN14437597, with the raw reads submitted to the SRA under accession number SRR12887722.
